# Ecological study of road traffic injuries in the eastern Mediterranean region: country economic level, road user category and gender perspectives

**DOI:** 10.1186/s12889-018-5150-1

**Published:** 2018-02-13

**Authors:** Mathilde Sengoelge, Lucie Laflamme, Ziad El-Khatib

**Affiliations:** 1 Department of Public Health Sciences, Karolinska Institutet, Widerströmska Huset, Tomtebodavägen 18 A, 171 77 Stockholm, Sweden; 2 University of South Africa, Institute for Social and Health Sciences, P.O. Box 1087, Lenesia, Johannesburg, 1820 South Africa; 3 South African Medical Research Council, University of South Africa’s Violence, Injury and Peace Research Unit PO Box 19070 Tygerberg, Cape Town, 7505 South Africa; 4 World Health Programme, Université du Québec en Abitibi-Témiscamingue (UQAT), Québec, Canada

**Keywords:** Road traffic, Mortality, Morbidity, Eastern Mediterranean region

## Abstract

**Background:**

The Eastern Mediterranean region has the second highest number of road traffic injury mortality rates after the African region based on 2013 data, with road traffic injuries accounting for 27% of the total injury mortality in the region. Globally the number of road traffic deaths has plateaued despite an increase in motorization, but it is uncertain whether this applies to the Region. This study investigated the regional trends in both road traffic injury mortality and morbidity and examined country-based differences considering on income level, categories of road users, and gender distribution.

**Methods:**

Register-based ecological study linking data from Global Burden of Disease Study with the United Nations Statistics Division for population and World Bank definition for country income level. Road traffic injury mortality rates and disability-adjusted life years were compiled for all ages at country level in 1995, 2005, 2015 and combined for a regional average (*n* = 22) and a global average (*n* = 122). The data were stratified by country economic level, road user category and gender.

**Results:**

Road traffic injury mortality rates in the Region were higher than the global average for all three reference years but suggest a downward trend. In 2015 mortality rates were more than twice as high in low and high income countries compared to global income averages and motor vehicle occupants had a 3-fold greater mortality than the global average. Severe injuries decreased by more than half for high/middle income countries but remained high for low income countries; three times higher for males than females.

**Conclusion:**

Despite a potential downward trend, inequalities in road traffic injury mortality and morbidity burden remain high in the Eastern Mediterranean region. Action needs to be intensified and targeted to implement and enforce safety measures that prevent and mitigate severe motor vehicle crashes in high income countries especially and invest in efforts to promote public, active transport for vulnerable road users in the resource poor countries of the Region.

## Background

The Eastern Mediterranean Region (EMR) consists of 22 countries in the Middle East, North Africa, the Horn of Africa and Central Asia and through its diverse number of countries it has the largest inequalities in the world [1]. Main challenges and threats for this region include climate change, water shortages and a heavy reliance on food imports, combined with population increase and unrest, civil conflicts, and wars in specific countries [2]. Huge health disparities exist across the region, which parallel disparities in socioeconomic development [3]. Rapid economic development and growth have led to increased motorization and improved road infrastructure for transport, but at a high cost [4]. The region has the second highest number of road traffic injury (RTI) mortality rates after the African region based on 2013 data [5], with RTIs accounting for 27% of the total injury mortality in the region [6]. RTIs were the ninth leading cause of disability adjusted life years (DALYs) for both men and women in 2013 and fifth for men only [2].

Globally the number of road traffic deaths has plateaued despite an increase in motorization, but it is uncertain whether this applies to the EMR. Country specific data in Oman [7] and the United Arab Emirates (UAE) [8] indicate higher mortality in 2016 compared to the year before. Recent data for the region and reliable long term trends are currently lacking, particularly data on non-fatal RTIs as morbidity data to date have had reliability and validity data issues [9]. In this study, the burden of RTIs was examined and potential differences within the region related to country economic development, mode of transport and gender. The following research questions were investigated:

1. What are the trends in RTI mortality and DALYs in the EMR region by country and income level compared to globally by income?
2. How do these trends differ by type of road user?
3. How do these trends differ according to gender?

The findings will be useful to guide the choice of interventions at regional and country level to reduce and prevent road injuries, as well as eliminate disparities between countries in the Region and in the EMR as a whole compared to other regions.

## Methods

### Design and data sources

A register-based ecological study was conducted at country level. Three publicly available databases were linked together in a three-step process. The first step consisted of extracting road mortality data at country level from the Global Burden of Disease (GBD) study 1995, 2005 and 2015 [10]. The EMR comprises the following 22 countries: Afghanistan, Bahrain, Djibouti, Egypt, Iraq, Islamic Republic of Iran, Jordan, Kuwait, Lebanon, Libya, Morocco, Oman, Pakistan, Palestine, Qatar, Republic of Yemen, Saudi Arabia, Somalia, Sudan, Syria, Tunisia, and the UAE. A total of 122 countries were identified with RTI mortality rates in the GBD study data expressed as number of deaths for all age groups combined and by gender. A full protocol detailing the data collection, treatment and estimation can be consulted elsewhere but in brief incompleteness, under-reporting, and misclassification corrections are individualized for each data source, country or subnational site, and year [10].

As the countries vary significantly in terms of their economic development they were stratified, in the second step, to three standard world economy groups according to the World Bank definition based on gross national income per capita: high income countries (HICs) at > US$ 12,616, middle income countries (MICs) at US$ 1036–12,615 and low-income countries (LICs) at < US$ 1035 [11]. LICs included Afghanistan, Djibouti, Yemen, and Somalia. MICs were Egypt, Iraq, Iran, Jordan, Lebanon, Libya, Morocco, Pakistan, Palestine, Sudan, Syria, and Tunisia. HICs included Bahrain, Saudi Arabia, Kuwait, Oman, Qatar, and the UAE.

The third step included extracting age and gender specific population data from the Population Statistics Division of the United Nations [12]. This was necessary as the GBD data provide no rates at regional or income level.

### Data treatment

RTI mortality rates and DALYs, per 100,000, were compiled for all ages at country level in ten year intervals (1995, 2005, 2015) and then combined for an EMR average (*n* = 22) and global average of EMR plus all other countries (*n* = 122). The results were then stratified by the three income levels, the type of road user and gender. The burden of road traffic deaths and injuries was presented through the compilation of two variables: a) average mortality rates per 100,000 with min and max values to provide the range as a measure of spread for road traffic deaths and deaths from other road injuries, for unprotected road users defined as pedestrians, bicyclists, motorcyclists and for protected road users defined as drivers of motor vehicles only; and b) DALYs as a measure of total burden, whereby one DALY represented one healthy year of life lost to road injury estimated by summing the fatal burden (years of life lost) and non-fatal burden (years of life with disability) due to road injury. A mortality gender ratio was compiled by dividing the mortality rate of males over the mortality rate of females; a DALYs gender gap was calculated by subtracting male DALYs from female DALYs. Data analysis was performed using Stata/SE 12.1 [13].

## Results

The EMR average RTI mortality rates were higher than the global average for all three reference years and all three income levels except for EMR MICs in 1995; see Table 1. At regional level there was a peak in 2005 and since a tendency for average mortality rates to decrease, similar to the global average (24.6; 26.7; 23.9 compared to 21.5; 21.0, and 18.4 respectively). Within income levels the differences between EMR countries and the global average is most remarkable in LICs, with rates consistently twice as high as the global rates but with a downward trend. Furthermore, there are large variations between the EMR countries for any reference year and country economic level, with very high mortalities in the LICs of Afghanistan and Yemen, MICs in Sudan and Iran, and HICs in Oman, Qatar, and UAE.

**Table 1:** Trend of road injury mortality all ages in the Eastern Mediterranean region (EMR) by country and income level compared to globally by income level

		Average road injury mortaliy rate per 100,000 (Min-Max)					
Income level	Country	1995	(Min-Max)	2005	(Min-Max)	2015	(Min-Max)
Low income	Low income countries	21.2	(17.3–25.7)	19.2	(15.9–22.9)	17.2	(14.2–20.8)
	Low income - EMR	42.4	(24.1–66.3)	40.9	(25.6–61.7)	38.5	(24.6–59.0)
	Afghanistan	54.0	(38.5–71.3)	54.6	(42.7–70)	49.9	(37.7–65.9)
	Djibouti	18.4	(10.9–31.3)	18.4	(9.4–33.4)	18.7	(9.3–36.5)
	Somalia	20.7	(8.1–42.4)	17.6	(6.8–38.4)	17.8	(6.9–39.9)
	Yemen	40.3	(21.1–63.9)	35.0	(20–55.7)	33.6	(20.4–53.7)
Middle income	Middle income countries	22.5	(20.6–25.0)	22.9	(21.0–25.0)	20.1	(18.3–22.3)
	Middle income - EMR	22.2	(16.2–30.4)	25.0	(18.4–33.6)	22.1	(15.7–31.1)
	Egypt	13.4	(12.3–14.6)	13.5	(12.7–14.3)	13.3	(12.2–14.4)
	Iraq	23.0	(18.3–28.4)	21.4	(16.8–27)	18.6	(13.8–24.7)
	Iran	36.9	(31.6–44.3)	50.7	(42.2–62)	39.0	(30.9–49.5)
	Jordan	20.0	(16–24.5)	14.4	(13.1–16.1)	12.8	(11.1–14.7)
	Lebanon	12.4	(9.6–15.6)	9.1	(6.5–13.2)	8.0	(5.4–12.1)
	Libya	21.4	(17.5–25.5)	22.6	(18.8–25.6)	21.2	(17.6–25.7)
	Morocco	22.6	(18.4–27.3)	18.7	(15.4–23.5)	18.5	(13.9–24.6)
	Palestine	13.3	(10.8–16.2)	11.7	(10.3–13.2)	12.1	(9.4–15.6)
	Pakistan	16.7	(13.3–22.1)	20.8	(16.8–27.3)	20.4	(15.8–27.4)
	Sudan	41.5	(25.3–63.4)	37.6	(24.3–55.4)	34.0	(22.6–50)
	Syria	13.8	(10.6–16.8)	11.8	(9.2–13.2)	10.8	(8.7–12.6)
	Tunisia	22.3	(19.2–25.7)	20.2	(16.8–23.7)	17.9	(14.2–22.7)
High income	High income countries	16.0	(15.5–16.4)	12.3	(11.9–12.5)	9.7	(10.1–9.4)
	High income - EMR	32.4	(26.0–39.6)	28.1	(23.5–33.1)	23.8	(16.9–25.4)
	Bahrain	19.8	(17.0–23.0)	15.9	(13.7–19.1)	11.1	(8.9–14.1)
	Kuwait	21.8	(20.8–22.9)	19.1	(18.2–20)	11.9	(10–14.5)
	Oman	52.7	(40.2–66.3)	40.8	(36.4–45.9)	40.5	(33.3–49.6)
	Qatar	44.8	(38.1–52.6)	40.9	(34.8–47.2)	28.4	(21.6–36.2)
	Saudi Arabia	29.9	(25.2–33.3)	26.4	(23.7–28.5)	23.6	(20.3–26.9)
	United Arab Emirates	40.1	(31.3–49.7)	34.6	(28.6–40)	35.2	(24.3–46.9)
Global average		21.5	(20.9–22.4)	21.0	(20.5–21.7)	18.4	(17.9–19.1)
EMR average		24.6	(17.4–34.1)	26.7	(19.4–36.2)	23.9	(16.7–33.5)

Table 2 shows that the distribution of road injury mortality rates across categories of road users is different between countries of the EMR and all countries globally. In all three reference years, protected road users had higher mortality than unprotected ones in the EMR (except for equal rates in MICs in 2005), whereas the opposite applies at the global level. Also, in each reference year and income level the mortality rates among protected road users in the EMR region is twice to three times that of all countries aggregated, with rates slightly increasing in EMR and slightly decreasing globally. From among the unprotected road users, pedestrians are by far the bigger risk group and the rates are quite similar between the income levels. The pedestrian mortality shows a downward trend in both EMR and globally. The difference between protected and unprotected road users in the EMR region is by far more pronounced in HICs followed by LICs and decreasing over time for all income levels. By 2015 the difference between the EMR average and global average for unprotected road users has narrowed to become similar for each type of unprotected road user.

**Table 2:** Trend of road traffic mortality rates in the Eastern Mediterranean region (EMR) and globally by type of road user and income level

		Average mortality rate per 100,000 (Min-Max)**					
Income level	Type of road injury	1995	(Min-Max)	2005	(Min-Max)	2015	(Min-Max)
Low-income							
EMR	Protected^	26.2	(13.7–33.7)	24.9	(14.5–31.8)	23.6	(14.8–28.9)
	Unprotected - average	**20.4**	**(13.6–35.7)**	**18.3**	**(12.4–32.3)**	**15.1**	**(10.5–26.0)**
	Pedestrian	16.1	(10.8–29.0)	14.2	(9.7–25.8)	11.6	(8.2–20.4)
	Bicycles	0.7	(0.4–1.2)	0.6	(0.4–1.0)	0.6	(0.3–1.0)
	Motorcycles	3.5	(2.3–5.5)	3.5	(2.3–5.4)	2.9	(2.0–4.6)
	Other road injuries	0.2	(0–0.6)	0.1	(0–0.4)	0.1	(0–0.3)
Low income countries	Protected^	9.2	(5.9–13.0)	8.0	(5.5–11.5)	7.6	(4.6–12.1)
	Unprotected average	**10.9**	**(6.8–16.3)**	**9.4**	**(6.2–13.8)**	**9.1**	**(5.3–15.1)**
	Pedestrian	8.7	(5.5–12.6)	7.3	(4.9–10.4)	6.9	(4.1–11.3)
	Bicycles	0.6	(0.3–0.9)	0.5	(0.3–0.8)	0.5	(0.3–1.0)
	Motorcycles	1.7	(0.9–2.9)	1.6	(0.9–2.6)	1.6	(0.9–2.8)
	Other road injuries	0.2	(0.1–0.5)	0.2	(0.1–0.4)	0.2	(0.1–0.4)
Middle-income							
EMR	Protected^	14.9	(11.4–18.3)	14.9	(11.7–18.2)	12.5	(9.8–15.6)
	Unprotected - average	**14.7**	**(10.5–20.8)**	**14.9**	**(10.8–20.5)**	**12.4**	**(8.9–17.7)**
	Pedestrian	10.0	(7.4–13.6)	9.3	(7.0–12.2)	7.7	(5.8–10.2)
	Bicycles	0.9	(0.5–1.6)	1.1	(0.6–1.9)	1.0	(0.5–2.1)
	Motorcycles	3.8	(2.6–5.5)	4.5	(3.2–6.4)	3.6	(2.5–5.5)
	Other road injuries	0.2	(0.1–0.4)	0.2	(0.1–0.4)	0.2	(0.1–0.4)
Middle income countries	Protected^	7.2	(5.7–9.8)	7.6	(6.2–9.5)	6.5	(5.1–8.2)
	Unprotected - average	**16.1**	**(12.6–19.7)**	**16.5**	**(13.9–19.3)**	**14.3**	**(11.6–17.4)**
	Pedestrian	11.8	(9.7–13.7)	10.7	(9.3–12.4)	9.0	(7.6–10.7)
	Bicycles	1.0	(0.7–1.3)	1.1	(1.0–1.3)	0.9	(0.7–1.1)
	Motorcycles	3.4	(2.2–4.8)	4.7	(3.7–5.6)	4.4	(3.3–5.5)
	Other road injuries	0.3	(0.2–0.5)	0.4	(0.2–0.5)	0.4	(0.2–0.5)
High-income							
EMR	Protected^	24.6	(20.5–28.9)	24.4	(21.3–27.5)	23.1	(17.9–28.5)
	Unprotected - average	**10.7**	**(7.6–14.7)**	**9.0**	**(7.2–12.5)**	**8.7**	**(5.7–13.9)**
	Pedestrian	8.6	(6.3–11.7)	7.1	(5.8–9.7)	6.7	(4.6–10.6)
	Bicycles	0.5	(0.3–0.6)	0.5	(0.4–0.7)	0.6	(0.3–1.1)
	Motorcycles	1.6	(1.0–2.4)	1.2	(1.0–2.1)	1.4	(0.8–2.2)
	Other road injuries	0.1	(0.0–0.1)	0.1	(0.0–0.1)	0.1	(0.0–0.1)
High income countries	Protected^	7.7	(6.9–8.3)	6.0	(5.5–6.5)	4.7	(4.3–5.4)
	Unprotected - average	**5.8**	**(5.2–6.8)**	**4.3**	**(3.8–4.9)**	**3.5**	**(2.9–4.0)**
	Pedestrian	3.6	(3.3–4.2)	2.7	(2.4–3.0)	2.2	(1.9–2.5)
	Bicycles	0.8	(0.7–1.0)	0.5	(0.5–0.6)	0.4	(0.3–0.5)
	Motorcycles	1.4	(1.2–1.6)	1.2	(1.0–1.3)	0.9	(0.7–1.1)
	Other	0.1	(0.0–0.1)	0.1	(0.0–0.1)	0.1	(0.0–0.1)
Global average							
	Protected^	7.5	(6.9–8.6)	7.4	(6.8–8.2)	6.3	(5.7–6.9)
	Unprotected - average	**13.5**	**(10.6–16.7)**	**13.7**	**(11.5–16.3)**	**12.2**	**(9.8–15.2)**
	Pedestrian	9.8	(8.0–11.6)	9.0	(7.7–10.5)	7.8	(6.4–9.6)
	Bicycles	0.9	(0.7–1.2)	1.0	(0.8–1.2)	0.8	(0.6–1.0)
	Motorcycles	2.8	(1.9–4.0)	3.8	(3.0–4.6)	3.6	(2.7–4.6)
	Other road injuries	0.2	(0.1–0.4)	0.3	(0.2–0.4)	0.3	(0.2–0.5)
EMR average							
	Protected^	16.6	(14.0–18.5)	16.6	(14.4–18.5)	17.5	(15.0–19.8)
	Unprotected - average	**15.0**	**(10.6–21.7)**	**14.8**	**(10.7–21.1)**	**12.4**	**(8.8–18.3)**
	Pedestrian	10.5	(7.7–14.9)	9.6	(7.2–13.4)	8.1	(6.0–11.3)
	Bicycles	0.9	(0.5–1.5)	1.0	(0.6–1.7)	0.9	(0.5–1.9)
	Motorcycles	3.6	(2.5–5.3)	4.2	(2.9–6.0)	3.4	(2.3–5.1)
	Other road injuries	0.2	(0.1–0.4)	0.2	(0.1–0.4)	0.2	(0.1–0.4)

*All ages aggregated; ^ it included motor vehicles only

**Fig. 1:**
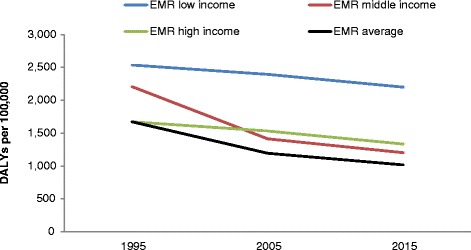
Change in road injury DALYs 1995–2015 by income level in the Eastern Mediterranean Region (EMR)

Fig. 1 shows the trend in DALYs by country income level. DALYS are decreasing in all country economies with a remarkable drop in MICs between 1995 and 2005 to reach the level of HICs. Both HICs and MICs remain lower than LICs.

**Fig. 2:**
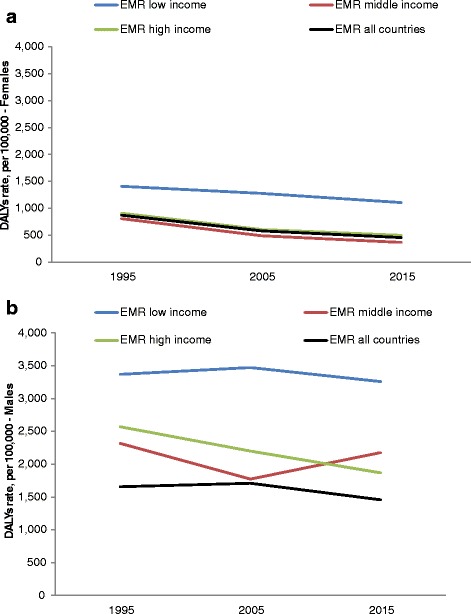
**a** Female road traffic injury DALYs 1995–2015 in the Eastern Mediterranean Region (EMR). **b** Male road traffic injury DALYs 1995–2015 in the Eastern Mediterranean Region (EMR)

When comparing DALYs by gender over time, the average female and male rates decreased from 1995 to 2005 and then rose again to pre-existing rates, with male DALYs more than three-fold compared to females (see Fig. 2 a and b). Stratified by income level, female and male DALYs for LICs are higher than for MICs or HICs for all three reference periods. In 2015, male DALYs in MICs are higher than for HICs, but LICs still 1.5 times higher; the same pattern is found for female rates in MICs.

## Discussion

### Country level differences

This study shows that RTI deaths in the Region are showing a tendency to decrease after 2005 but remain consistently higher than the global average. Large inequalities exist within the region in both deaths and injuries. Stratification by country income level shows that the LICs carry the largest mortality and morbidity burden for all three reference periods, with a downward mortality trend but minimal decrease in morbidity compared to the MICs and HICs. EMR motor vehicle occupants show an increasing trend and in 2015 are approximately 3-fold higher on average compared to globally. This contrasts to unprotected road user mortality that decreased over time and is now similar to global rates, overall and disaggregated as well.

Countries from the Region are collectively facing considerable road safety challenges. The first one is related to engineering – i.e. the organization and design of the road infrastructure and that of the vehicles on the road, which influence the likelihood of crashes to occur and also the consequence severity [14, 15]. According to WHO data there are large variations between countries in the Region related to legislation to support road safety; for example, only two countries (Morocco, Qatar) have developed national policies and enabling environments to separate unprotected road users from high speed traffic and one-third of countries do not inspect roads [5]. Afghanistan scores lowest out of all countries in the Region in all categories of road safety legislation (e.g. no road safety strategy, no motorway speed limit, practically no enforcement of the speed limit law or drink driving law, etc.), which combined with its mountainous terrain are factors that may relate to this country having the highest road traffic deaths and disabilities in the Region. Yemen has a road safety strategy but it receives no funding and has no fatality reduction target and there is zero enforcement of speed limit or drinking and driving laws, which may be contributing factors to explain its high rates [5]. As this is an ecological study it was not possible to identify the direct causes of the RTIs in this very heterogeneous road user population made up of different backgrounds, driving cultures and training systems. Crash data at the individual level point to a myriad of causes, such as poorly designed or maintained roads, older or unsafe vehicles (overcrowded minibuses, Tata buses), lack of footpaths and poor visibility at night [16]. Secondly, there are also issues related to road safety behavior, with high levels of risk taking among various categories of road users and poorly developed and enforced safety regulations [17–20]. This includes speeding, responsible for about 50% of RTI deaths globally [21] and also a significant factor in the EMR region. In addition, local studies found a seatbelt non-compliance rate ranging from 48% [22] to 82% [23] in the total road traffic crashes and 58% non-compliance when self-reported [24]. This is despite the fact that the majority of the EMR countries reporting the existence of national legislation in national speed limits, drink-driving, motorcycle helmet, seat-belt, drug-influenced driving and mobile phone use (distracted driving) laws [5]. Thus, consistent and effective enforcement of safe behavior on the road is in need of greater attention in all countries in the Region [25].

A third set of explanations – and avenues to reduce the high rates observed – has to do to with the paucity of alternative and collective modes of transport. This is particularly important in the MICs that are struggling to manage the unprecedented economic growth leading to increased urbanization, motor vehicles and road construction without the necessary investment in road safety management [4]. Clearly, transportation in general has been disrupted by the civil unrest and violent conflict in the Region, although little is known about how this is impacting road safety, including provision of ambulance services. The conflict is occurring exclusively in the LICs and MICs of the Region. These same countries face a disproportionate burden in RTIs. More than half of the countries in the Region are currently facing either acute or chronic crises with health systems facing major challenges in coping with the protracted emergencies [26]. Only one published study could be identified that reported on the impact of the armed conflict in Benghazi, Libya on road injuries. The study found there were fewer RTIs during the conflict period but a higher morbidity and mortality in those that did occur [27]. Other regional research shows there is a worsening in health conditions overall, including a decrease in life expectancy for Syrians in particular [2], and an accelerating rate of both premature mortality and nonfatal outcomes due to mental health issues matching the increased level of instability in the Region [28].

### Category of road user and gender base differences

In the EMR protected road users are consistently at higher risk of fatal RTIs compared to unprotected. Within the region in 2015 their mortality rate is almost three-fold that of unprotected road users in HICs, 1.5-fold in LICs and similar only in MICs (still double global average). As indicated above, these rates surpass global ones as unprotected users have double the risk of fatal RTIs on average globally. Pedestrian deaths drive the rates of unprotected road user deaths in the EMR which mirrors the global average as pedestrians account for 35% of road injury deaths globally [16]. A study in Al Ain in the UAE found that 88% of the pedestrian injuries occurred in young non-nationals and the peeks in increased injuries coincided with time shifts of construction workers [29]. This suggests a socioeconomic inequality in vulnerable road user mortality, which may exist in other countries in the Region.

As is the case worldwide, RTIs are also unevenly distributed between males and females. Mortality and morbidity rates are higher for males compared to females which reflects the global picture [16]. The most common reasons lying behind those differences are related to differences in levels of exposures, with men being on the road to a far greater extent than women, and in road safety behaviours, with men being far more inclined to take risks/adopt risky behavior than women. Differences in exposures between men and women find an explanation in regional legislations and practice. Women driving in Saudi Arabia is not against the law since September 2017 but they still need power of attorney from a male relative to acquire a car; in other countries women are prohibited from driving by their families [30, 31]. Thus, those allowed to drive have been driving shorter periods of time than men, drive less and are less likely to drive long distances or on the highway where high speed crashes occur. There is no published data demonstrating whether EMR women are less reckless drivers (jumping red lights, illegal U-turns, aggressive driving) than men. There are few studies from the Region examining this and one study points to surprisingly very small differences in self-reported risk behaviours between women and men [17].

### Study limitations

The ongoing unrest and war may have affected the quality of the health data in the EMR, as some GBD data points were not available and modelling techniques were used to generate the estimates based on other available variables or data from neighbouring countries or countries with a similar health profile in the Region [2]. Regarding the quality of the road data specifically, a comparison between GBD 2010 road injury mortality estimates and government statistics from high-income country data in the International Road Traffic Accident Database found an overestimation in the GBD estimates due to an excess of partially specified causes of deaths being attributed to road injuries [32]. Also, rates may be underreported for lower or middle income countries as official statistics were found to account for less than 60% of estimated fatalities in these countries and few have a specific traffic surveillance system [33, 34]. Furthermore, all unprotected road users are collapsed in one category that is mainly composed of pedestrians, as other categories of unprotected road users remain low in the Region.

### Implications

Our findings suggest that road safety strategies need to be intensified in order to maximize lives saved in the EMR; particularly for protected road users in HICs and LICs as well as unprotected in LICs. Yet the essential requirements of connectivity, affordability, security and ability to access important domains like education, health and market are shared by all EMR countries. A shift of focus is necessary in transport policies to promote public and active modes of transport for improved health and reducing vehicle emissions [35, 36]. Also, meeting the mobility needs of the low-income population should be reprioritized and incorporated as an essential service of welfare policies [37]. System-wide investments in vehicle quality, enforcement, safe infrastructure, and analyses of road users in the pre-crash, in-crash, and post-crash stages are necessary in order to build and manage transport systems that are safe, clean, and affordable for all road users in the Region [38–40].

## Conclusions

Road safety continues to be a priority in the EMR as road traffic mortality and morbidity remains high in the EMR compared to globally and the suggested mortality downward trend over the past 20 years is minimal. The inequalities within the Region suggest that all of these countries, regardless of income level, need to intensify the implementation and enforcement of safety measures to prevent and mitigate severe crashes. These include implementation of a speed enforcement strategy, initiating a shared responsibility towards road safety, revising traffic laws, increasing ambulance services and supporting road safety research to evaluate which countermeasures work best given the diverse settings found in the Region. Such investments are essential in order for the EMR as a whole to accelerate progress towards achieving the road safety targets of the Sustainable Development Goals by 2030.

## Abbreviations

EMR: Eastern Mediterranean Region
GBD: Global burden of disease
HICs: High income countries
LICs: Low income countries
MICs: Middle income countries
RTI: Road traffic injuries
UAE: United Arab Emirates
